# Automation of Polysaccharide Quantification: A Rapid High-Throughput Assay Enabled by Liquid Handling Technology

**DOI:** 10.3390/biotech15010024

**Published:** 2026-03-20

**Authors:** Samuel Nicacio, Winston Umakanth Balasundaram, Aboli Bhingarkar, Daniel Cho, Rashmi Ghayal, Anup Datta, Subhash V. Kapre

**Affiliations:** 1 Quality Control, Inventprise, Inc., Redmond, WA 98052, USA; snicacio@inventprise.com (S.N.); winston@inventprise.com (W.U.B.);; 2 Bacterial Research and Development, Inventprise, Inc., Redmond, WA 98052, USA; 3 Inventprise, Inc., Redmond, WA 98052, USA

**Keywords:** anthrone, pneumococcal, polysaccharide, automation, validation

## Abstract

Different methods are used today for polysaccharide quantitation, including HPLC and various colorimetric assays. Among these, the anthrone-sulfuric acid assay (anthrone assay) is popular when the sample matrix is suitable, such as in purified polysaccharides and monovalent bulk conjugate components of glycoconjugate vaccines. While relatively safe, quick, and affordable, the anthrone assay requires significant operator time to complete and is not suited to high-throughput processing. Furthermore, the anthrone-sulfuric acid reagent presents a unique challenge to automation efforts due to its corrosive properties. Reported here is an automated anthrone assay via a liquid handling system (LHS). Twenty-three serotypes of pneumococcal (PNU) polysaccharide were quantified with the traditional anthrone assay and subsequently analyzed using the anthrone LHS method. The anthrone LHS method was evaluated for accuracy compared to the manual method and later validated according to ICH Q2 (R2) guidelines. To our knowledge, this is the first fully unattended and corrosion-mitigated anthrone assay validated under ICH Q2 (R2), capable of overnight batch operation. The developed assay can quantify polysaccharides with an accuracy of 81–115%, is precise to a coefficient of variation of <7.0%, and is linear between 30 and 650 µg/mL range (R^2^ ≥ 0.993). The assay can process eight samples per hour, can be utilized in overnight operation, and completes all pipetting, incubation, and data export steps automatically.

## 1. Introduction

Colorimetric assays are widely accepted as standard reference tests for polysaccharide quantification in glycoconjugate vaccine development and characterization [1]. Their relatively low cost and time to complete compared to HPLC methods make them especially desirable in situations requiring high-throughput analysis [2,3]. Among these, the anthrone-sulfuric acid assay (anthrone assay) is widely used where possible, as it is less hazardous than other phenol-based methods, even if it is less sensitive [4]. It has been seen to have wide application across fields of plant biology, medicine, and microbial research, wherever polysaccharides may be found, given some sample restrictions [5,6,7,8,9,10,11]. The limitations of the anthrone assay are due to the reagent’s sensitivity to many interfering compounds and the requirement for the presence of hexose sugars in the sample to test [12,13]. In testing polysaccharide and monovalent bulk conjugate intermediates for saccharide content, the anthrone assay remains the safest, quickest, and least expensive option available. Further reducing assay completion time is important as these in-process materials are the bottleneck for glycoconjugate vaccines [14]. Each serotype involved in the vaccine requires its own production, purification, and characterization steps [2,14].

Regardless of the method used, colorimetric quantification of polysaccharides often relies on hydrolysis and dehydration to prepare the polysaccharide for binding to the staining reagent, typically at highly acidic conditions [13,14]. The resulting compounds are then ready for determination via spectrophotometry. The sulfuric acid used by the anthrone assay is highly corrosive, and significant precautions should be exercised during its use. This reaction is typically performed in a hot water bath, either in glass vials or plastic tubes. The time needed for the reaction ranges from 10 to 20 min depending on the method, and this assay requires significant attention and manual input from the analyst to complete [15,16,17]. Additionally, multivalent vaccines requiring polysaccharide testing of multiple serotypes create additional strain on the scalability of the assay, as standards are peculiar to their serotypes [14]. Because of this, samples can only be batched if they are of the same serotype. These challenges highlight the need for an automated microplate-based approach suitable for high-throughput polysaccharide analysis in glycoconjugate and polysaccharide vaccine manufacturing.

Some have improved on the traditional form of the assay by performing the incubation reaction directly in a microtiter plate [15,16,17]. These changes simplify and speed up the process, though they still involve the manual handling of sulfuric acid. This manual sample and hazardous reagent handling may be automated by the use of a liquid handling system (LHS). Liquid handling systems have undergone significant development and adaptation in recent times, with some even re-purposing 3D printing technology as an LHS [18]. Positive displacement LHSs may provide superior control for hazardous reagent pipetting, while air displacement LHSs appear to find more widespread use [19,20,21,22,23]. Any efforts to automate the anthrone assay within a liquid handling system (LHS) are largely unreported, with one exception [24].

Turula et al. employed the use of a Janus LHS, leveraging the microtiter improvements mentioned for an automation version of the anthrone assay [24]. Turula et al. describe an automated method from loading the sample, reagents, and labware to reading on a spectrophotometer. After the reading, the LHS manifold is wiped with water to prevent sulfuric acid corrosion over time. This method addresses variability from operator to operator through automated pipetting and greatly reduces the hands-on time needed to perform the experiment. While a significant improvement, their process is limited in the application of one anthrone test at a time, as well as additional wiping steps to maintain the system. Our proposed method can batch multiple anthrone tests together, does not require any manual wiping/maintenance specifically for sulfuric acid corrosion, and is validated for GMP use.

The objective of this study was to design and validate a fully automated anthrone assay using an LHS capable of processing large polysaccharide batches with minimal manual intervention and corrosion risk from reagents. The method described here uses the Hamilton STAR LHS and has the capability to handle multiple plates in succession, allowing for overnight/weekend operation. Anthrone-sulfuric acid reagent is securely stored in a custom reagent container, negating the need for washing or maintenance steps due to the reagent. Any errors not automatically resolved during method execution are escalated to the performing analyst via email or are visible via remote connection to the computer attached to the LHS. This method processes up to eight samples per hour, and can complete up to 60 samples (five serotypes) before needing additional analyst input. The assay range has been demonstrated as linear, precise, specific and accurate for 23 different PNU serotypes according to ICH Q2 (R2) guidelines [25].

## 2. Materials and Methods

### 2.1. Anthrone-Sulfuric Acid Reagent

**Figure 1:**
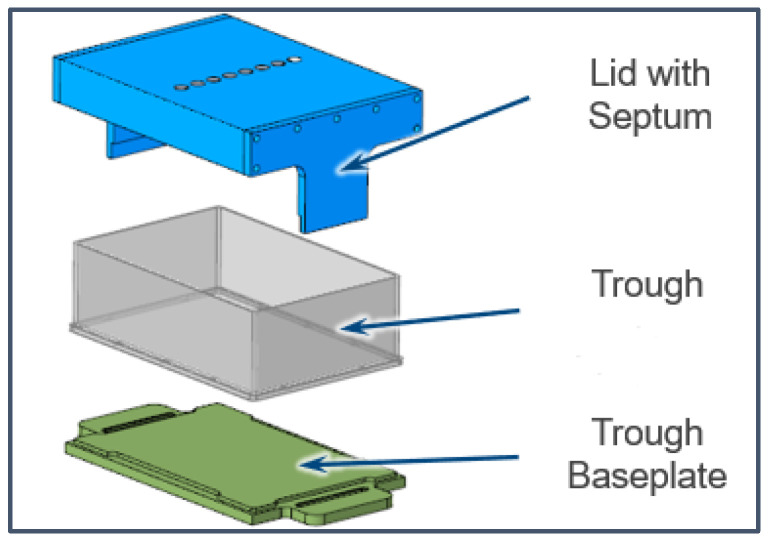
Diagram of hazardous reagent housing apparatus designed to limit corrosion effects of anthrone reagent on the rest of the liquid handler.

The anthrone-sulfuric acid reagent was prepared by mixing 0.2 g anthrone reagent (97%) with 100 mL sulfuric acid (>96%) for 15 min (Merck, St. Louis, MO, USA), and was chilled on ice for at least 15 min. During LHS operations, the reagent was transferred to a 200 mL reagent reservoir (Nalgene, New York, NY, USA) and housed in a custom hazardous reagent container (Hamilton, Reno, NV, USA); concept pictured in Figure 1. The container houses a 300 mL reagent trough and covers the entire opening with a rubber septum and metal plating with holes for LHS pipettes. The rubber septum is cut underneath the holes in the metal plate to allow pipette tips to pass through, but naturally closes when not in use. When performing the assay manually, it was accessed directly from the beaker. The anthrone-sulfuric acid reagent was prepared fresh on the day of use and stored at 4 °C before use.

### 2.2. Manual Anthrone Assay

PNU 4 and 5 Standards (SSI Diagnostica, Radford, VA, USA, articles 76855, 76857, respectively [26]) were diluted to a range of 10–650 µg/mL, and all other serotypes were diluted to 10–220 µg/mL. 300 µL of each level was transferred to 1.5 mL tubes and 600 µL of anthrone-sulfuric acid reagent was transferred to the tubes via a repeater pipette (Eppendorf, Framingham, MA, USA) for a final tube volume of 900 µL. Samples were vortexed briefly and then placed in a water bath at 90 °C for 12 min, followed by an ice bath incubation for 3 min. The samples were then transferred to a 96-well plate in triplicate (Costar, Phoenix, AZ, USA) and read via plate spectrophotometer at 625 nm (Agilent, Santa Clara, CA, USA).

### 2.3. Anthrone LHS Assay

After transferring stock, standard, sample, and reagents to the liquid handler deck, all LHS operations presented were executed by a Hamilton STAR liquid handling system (Hamilton Company, Reno, NV, USA). All liquid handling steps were executed using pre-validated Hamilton STAR default liquid classes without custom viscosity tuning. This system contains an Epoch spectrophotometer operated by Gen5 software v3.16 (Agilent, Santa Clara, CA, USA). The software controlling the Hamilton STAR (Venus) controlled Gen5 to operate the spectrophotometer, so no manual manipulation was needed to use the spectrophotometer.

All tips used by the LHS were Hamilton brand conductive tips, and the formats used were 50 µL filtered, 300 µL nested, 1000 µL filtered, and 300 µL SlimTip filtered tips. Standards were obtained from SSI Diagnostica, and samples were purified polysaccharides manufactured in-house at Inventprise. Both standard and samples were diluted in 5 mL dilution tubes (Globe Scientific, Mahwah, NJ, USA) in water for injection (WFI, Cytiva, Logan, UT, USA). 80 µL of standard and sample were transferred to respective wells in a 96-well microplate in triplicate. After chilling the plate for 3 min in a cold chest, 160 µL of the anthrone-sulfuric acid reagent was pipetted to each well in the plate containing material, followed by a 40 min incubation on the heater-shaker set to 90 °C. After the incubation, the plate was incubated at room temperature for 4 min, and then chilled for 5 min in the cold chest.

**Figure 2:**
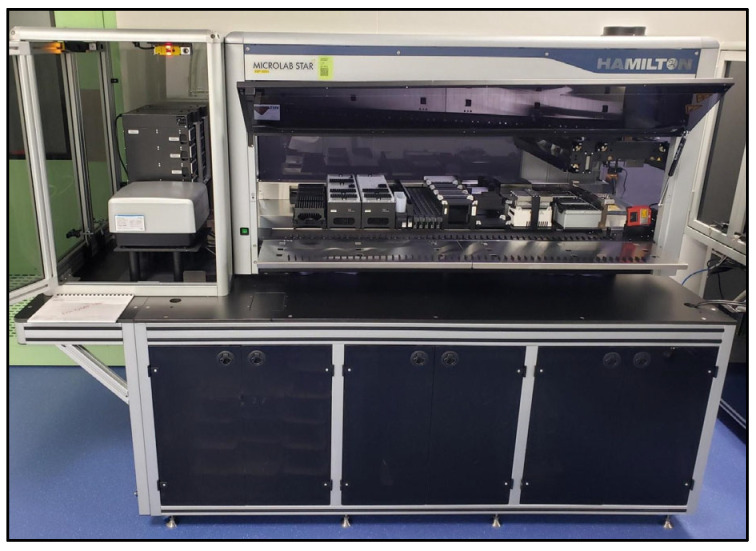
Liquid Handling System used for anthrone LHS testing.

The plate was then transferred to the spectrophotometer and read at 625 nm via a pre-determined Gen5 protocol, and exported as a csv for LIMS integration. Excel was used in development when many plate layout changes were being made in the optimization process. The entire LHS is pictured below in Figure 2.

## 3. Results

### 3.1. Comparability Testing

**Figure 3:**
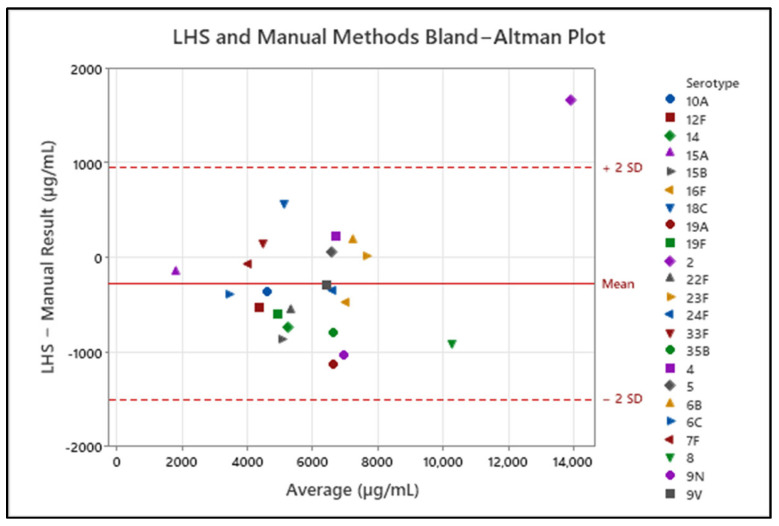
Bland–Altman plot of results obtained by both LHS and manual methods. The difference in analytical result of the two methods is compared to the average of the two methods. The solid red line represents the mean of the differences, and the dotted lines represent ±2 standard deviations from the mean.

Comparability testing was performed on 23 PNU serotypes of purified polysaccharide: 2, 4, 5, 6B, 6C, 7F, 8, 9N, 9V, 10A, 12F, 14, 15A, 15B, 16F, 18C, 19A, 19F, 22F, 23F, 24F, 33F, and 35B. Each sample was assessed via manual anthrone assay and then tested via the LHS. Comparability was determined by the ratio of the LHS result of a serotype to its manual result, and is expressed as a percentage in Table 1. A ±20% comparability criterion was selected in alignment with typical acceptance ranges for colorimetric carbohydrate assays used in early-stage and in-process control testing. All serotypes conform to this definition of comparability. Additionally, for each serotype, the difference in results obtained between the LHS and manual methods was assessed via a Bland–Altman plot, in Figure 3. All serotypes except PNU 2 fall within ± 2 standard deviations of the mean.

### 3.2. Edge Effects

**Figure 4:**
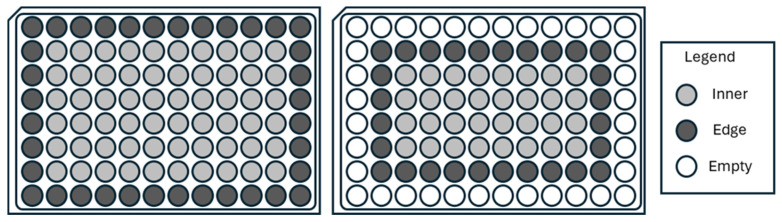
Plate map for edge effect investigation.

Because the anthrone incubation step is being performed directly in the 96-well plate on a plate heater, it is necessary to assess the uniformity and consistency of the incubation. The wells located along the perimeter of the plate are typically the most susceptible to inconsistencies, as they may be located close to the edge of the plate heater. Differences in incubation between the edge of the plate and the rest of the plate are called edge effects. To evaluate any potential edge effects on the plate, the absorbance of the purified polysaccharide sample was measured across the plate in two configurations. One plate contained samples in all 96 wells, another contained samples only in wells not on the perimeter of the plate (inside 60), with the perimeter wells empty. Comparison schemes between perimeter wells and inner wells are outlined in Figure 4, and *t*-test results are reported in Table 2.

In both cases, small but statistically significant differences exist between perimeter and inner wells on the hot plate. The magnitude of the difference (<2%) was not considered analytically, and statistically significant due to the high amount of replicates. While these differences are very low, certain perimeter wells, such as A1 and H1, display lower absorbances, indicating greater than 5% discrepancies compared to the rest of the plate. Validation of the assay will proceed with the interior 60 wells used only, as all absorbances across the plate run in this condition do not exceed ±5% the average absorbances, indicating a stable response across this area and minimal potential warping due to prolonged exposure to the 90 °C hot plate. Analysis of potential plate warping by this method is consistent with the approach taken by Turula et al. [24].

### 3.3. Anthrone LHS Validation

To validate the anthrone LHS method for PNU polysaccharides, seven representative serotypes (2, 4, 5, 6B, 8, 9V, and 12F) were chosen to be assessed for specificity, precision, linearity, and accuracy, according to ICH Q2 (R2) [25]. Validating the anthrone LHS method for each representative serotype would show the assay is suitable for quantitation for 23 PNU serotypes: 2, 4, 5, 6B, 6C, 7F, 8, 9N, 9V, 10A, 12F, 14, 15A, 15B, 16F, 18C, 19A, 19F, 22F, 23F, 24F, 33F, and 35B. Criteria and their acceptable ranges are shown in Table 3.

Polysaccharide sample was pipetted at various concentrations by the liquid handler across the range of the standard curve. During each assay, samples were diluted independently 12 times (biological replicates) and were plated in triplicate (technical replicates). Intra-assay coefficient of variation was calculated by dividing the standard deviation by the average of all biological replicates in an assay, reported in Table 4. This was repeated twice for a total of three assays, and the inter-assay coefficient of variation in all biological replicates is reported in the “Overall” column reported in Table 4. Sample replicates were plated to different parts of the plate to include any plate bias in the results.

Linearity was assessed by serially diluting the sample 2-fold across the range of the standard curve, and taking the coefficient of determination from the linear regression. Three assays were performed, and the coefficient of determination for all assay data for each serotype is presented in Table 5. Accuracy was calculated by dividing the observed concentration of a sample by the theoretical concentration for that sample. Accuracy was calculated for each level of the linearity data, and the range of accuracies is also presented in Table 5.

## 4. Discussion

The efforts documented here were targeted at establishing the anthrone LHS assay to be comparable to the assay performed manually. This was evaluated in two ways. First, the anthrone LHS assay should achieve a comparable result to the manual assay as performed for a given sample. The primary acceptance criterion for comparability was LHS results within ±20% of the manual result. While all serotypes passed this criterion, 21 of the 23 serotypes were within ±15% of their respective manual results, representing a typical performance of the LHS method. Second, the anthrone LHS assay itself must pass all validation criteria according to ICH Q2 (R2) for precision, linearity, specificity, accuracy, and range. This is also shown.

The physical incubation of the 96-well plate via the LHS hot plate was assessed for inconsistencies within and at the edges of the plate. While the difference between the outer and inner wells of the plate was small compared to the average absorbance observed, the incubation proved more consistent when the outside wells were left blank. For the sake of time, validation of the anthrone LHS assay proceeded with keeping the outside wells blank. This decision was considered a trade-off given the dramatic increase in throughput the LHS method provides. It would be worthwhile to revisit validation of the assay using all wells on the plate, with special attention given to the linearity and precision results.

The chemical differences between the serotypes necessitate unique polysaccharide standards for suitable quantitation. Unique serotypes also present differences in viscosity. For the liquid handler to accurately pipette a unique liquid, various factors like pipetting speed, air cushion volume and pre-wetting cycles, among others, must be closely tuned. These parameters are stored in a computer file called a liquid class. The Hamilton STAR comes equipped with standard liquid classes for routine use, but Hamilton recommends liquid class development for unique samples to guarantee liquid pipetting accuracy expected during preventative maintenance (typically within ±5%). Instead of performing this time-intensive work, our approach here was to dilute each sample 2-fold as the first step in the method. This mitigated variance due to differing viscosity among the serotypes and allowed one LHS method to apply to all serotypes. While we did not achieve less than 5% across all measured precision values (likely due to not creating custom liquid classes at this time), all validation criteria were satisfied, and this prevented the need to make and maintain copies of the method to accommodate different liquid classes.

An important consideration raised by Turula et al. is the potential for corrosion of the LHS from the anthrone-sulfuric acid reagent [24]. In that study, the manifold of the liquid handler was washed with water after sample analysis to prevent this. To avoid this level of maintenance, a custom hazardous reagent container was created by Hamilton engineering. This container was used in LHS operations to securely house the anthrone reagent. It should be noted that the Hamilton STAR is not designed to use this reagent, that doing so may void our service agreement, and our use of this reagent was performed at our own risk. The Hamilton STAR used in this study received bi-annual preventative maintenance from the Hamilton company for 3 years, with no corrosion damage to the instrument found. 

The cycle time of the assay, as validated, is about 80 min, depending on the time it takes for the user to initialize the assay. This time involves incubating the plate for the anthrone incubation for 40 min to allow full decomposition of the polysaccharide sample for the anthrone reaction. This increased time needed for the highest degree of linearity in the standard curve is likely due to a relatively inefficient heat transfer from the hot plate when compared to the water bath, resulting in incomplete reaction of the high concentration polysaccharide when incubated for 12 min only.

After validation, the sample reagent ratio and the incubation time were revisited. The amount of sample per the amount of reagent was further decreased until the ratio was 1:5 instead, by pipetting 40 µL of sample and 200 µL of reagent. In doing so, it was observed that even an incubation time of 20 min will likely satisfy validation requirements for linearity, with comparable results, at the cost of decreased absorbance overall. Further testing of different PNU serotypes should consider a sample reagent ratio of 1:4 or 1:5, and an incubation time of 20 min. This would bring the cycle time for the assay to just over an hour and would be a profitable post-validation optimization exercise.

Regardless of the cycle time, the solution presented here allows the user to obtain data in LIMS from an anthrone assay in less than an hour and a half. As validated, the assay can complete and analyze up to 12 samples in the 90 min assay run time. When a protocol was predefined in Gen5, data analysis and export can happen automatically after the plate is read. The user is required to confirm dilutions used as well as sample metadata, but all other analysis is performed and securely exported via csv to a data repository for integration to a LIMS or other data processing pipelines. This data pipeline from a spectrophotometer to a LIMS was developed for Inventprise’s use in the QC lab.

The capacity to automate and prepare multiple plates without further user input allows this method to operate overnight. To support this, the method solicits sample information from the user for each serotype requiring analysis. All the anthrone-sulfuric acid reagent and WFI are prepared ahead of time, and the user pre-stacks the LHS with additional labware. The method is programmed to send an email to a user if any step in the process is unable to proceed, limiting any downtime in case of an event.

While the method was validated for PNU polysaccharides, the work needed to adapt the method to a different chemistry is minimal. Other PNU serotypes should be evaluated to see if their chemistries fall under the general patterns of the others presented here; if so, there may be a rationale to simply apply this assay to those. Otherwise, the assay will need to be adapted. The hazardous reagent container is suitable for housing other materials, and the liquid class of the transfer steps for the hazardous reagent can be changed to a different preset to accommodate volatile reagents or those with different viscosities. The incubation conditions can be changed, including placing the plate in the dark for the incubation instead. Pipetting steps are robust and accommodate any dilution factor between 2 and 400x (Neat pipetting is possible for liquids of typical viscosities). The largest consideration would be reprogramming the standard curve steps, as levels in the curve are explicitly defined. By looking at these parameters, other colorimetric polysaccharide quantitation assays, such as resorcinol, uronic acid, phenol, and 2-phenoxyethanol, should be suitable for automation with this LHS method.

## 5. Conclusions

High-throughput application of the anthrone assay for polysaccharide quantitation is hampered by the need for manual input throughout the process, the corrosive nature of the anthrone-sulfuric acid reagent, and the harsh conditions of the incubation process. Reported here is a fully validated automated anthrone assay that overcomes these barriers.

Taking the anthrone assay from a manual process to this automated platform should dramatically increase the throughput of polysaccharide quantitation in glycoconjugate vaccine manufacturing. The assay is capable of overnight operation and allows an operator to complete up to eight samples per hour without special maintenance to prevent corrosion from the anthrone-sulfuric assay. This assay is ready for use in the IVT QC lab and is suitable in GMP/CMC-regulated spaces. The developed assay can quantify polysaccharides with an accuracy of 81–115%, is precise to a coefficient of variation of <7.0%, and is linear between 30 and 650 µg/mL range (R^2^ ≥ 0.993). The export of data directly to LIMS allows a validated approach from sample loading through data analysis.

## Figures and Tables

**Table 1:** Comparability of PNU polysaccharides between LHS and manual methods ^1^.

Serotype	Manual Result (µg/mL)	LHS Result (µg/mL)	Accuracy (%)
2	13,094	14,763	113
4	6605	6830	103
5	6532	6594	101
6B	7140	7316	102
6C	3607	3212	89
7F	4063	3994	98
8	10,747	9836	92
9N	7462	6425	86
9V	6576	6284	96
10A	4760	4400	92
12F	4602	4076	89
14	5588	4848	87
15A	1879	1729	92
15B	5472	4607	84
16F	7279	6809	94
18C	4849	5419	112
19A	7173	6048	84
19F	5227	4634	89
22F	5598	5032	90
23F	7609	7619	100
24F	6807	6454	95
33F	4391	4543	103
35B	7033	6245	89

^1^ Samples were analyzed manually and via the anthrone LHS method with three biological replicates, and the average was reported. Except for PNU 15B and 19A, all serotypes performed within ±15% between the two different methods, indicating this as typical behavior of the assay. All serotypes satisfy the primary criterion of a maximum difference of ±20% between the two methods.

**Table 2:** Coefficient of intra-assay and inter-assay variation in polysaccharides using the LHS method. Assays were performed on different days by different analysts.

Test Condition	Inner Wells Average Absorbance	Edge Wells Average Absorbance	% Difference	P (t ≤ T)
All wells	0.88	0.87	−1.7%	2.9 × 10^−6^
Perimeter empty	0.80	0.80	0.8%	0.011

**Table 3:** Criteria and acceptable ranges for samples during validation from ICH Q2 (R^2^).

Criterion	Measured Value	Lower Limit	Upper Limit
Specificity	Expected/Observed	80%	120%
Precision (Intra-Assay)	Coefficient of Variation (CV)	-	5%
Precision (Inter-Assay)	Coefficient of Variation (CV)	-	10%
Linearity	Coefficient of Determination (R^2^)	0.99	-
Accuracy	Expected/Observed	80%	120%

**Table 4:** Coefficient of intra-assay and inter-assay variation of polysaccharides using the LHS method, presented as CV. Assays were performed on different days by different analysts.

Serotype	Assay 1 (%)	Assay 2 (%)	Assay 3 (%)	Overall (%)
2	2.9	3.9	4.2	7.0
4	3.9	2.7	2.8	3.5
5	4.2	3.4	2.9	3.7
6B	2.5	2.9	2.5	3.1
8	2.4	3.2	2.0	3.5
9V	2.9	2.1	1.4	2.8
12F	1.7	1.9	2.5	2.8

**Table 5:** Linearity, specificity, and accuracy of PNU polysaccharides during validation.

Serotype	R^2^ Value	Specificity (%) ^1^	Accuracy (%)
2	0.9984	100–102	87–105
4	0.9930	101–105	95–104
5	0.9985	95–96	85–104
6B	0.9998	99–105	88–104
8	0.9982	99	81–115
9V	0.9989	97–101	82–103
12F	0.9993	96–102	81–107

^1^ Specificity was evaluated by spiking the sample with a polysaccharide standard, and spiking another aliquot of the sample with potentially interfering compounds found in the matrix: BSA and residual DNA. Both spike results were divided by their theoretical values, reported as a percentage. Accuracy values represent the lowest and highest recoveries obtained across the standard curve.

## Data Availability

The original contributions presented in this study are included in the article. Further inquiries can be directed to the corresponding author.
